# 

**Published:** 1888-03

**Authors:** 


### THE BRISTOL

### Medico-Chirurgical Journal.

A JOURNAL OF THE MEDICAL SCIENCES FOR THE
WEST OF ENGLAND AND SOUTH WALES

*PUBLISHED UNDER THE AUSPICES OF*

*THE BRISTOL MEDICO-CHIRURGICAL SOCIETY.*

EDITOR:
J. GREIG SMITH, M.A., F.R.S.E.

ASSISTANT-EDITOR:
L. M. GRIFFITHS.

---

"Scire est nescire, nisi id me

Scire alius sciret."

---

25-1

SURGEON GEN'L'S OFFICE

122912

LIBRARY

*VOL. VI.*

BRISTOL: J. W. ARROWSMITH;
LONDON: J. & A. CHURCHILL, 11 NEW BURLINGTON STREET.
1888.

WI
BR323

J.W. ARROWSMITH,

Printer and

Publisher,

QUAY STREET, BRISTOL.

### CONTENTS OF VOLUME VI.

ORIGINAL PAPERS :— *Page.*

Droitwich and its Brine Baths. By ALFRED CRESPI ... ... ... 1

The Treatment of Incomplete Abortion. By A. E. AUST LAWRENCE, M.D. ... ... ... ... ... ... ... ... ... ... ... 8

Significance of Hoarseness and Aphonia in cases of Pulmonary Phthisis. By BARCLAY J. BARON, M.B. ... ... ... ... 12

Menorrhagia, etc., and Hygienics. By JOHN MEREDITH, M.D. 17, 102

Intubation of the Larynx. By JOHN DACRE.... ... ... ... ... 73

Notes on Disinfection. By D. S. DAVIES, M.B. ... ... ... ... 83

The Medical Certificate of the Cause of Death. By AUGUSTIN PRICHARD ... ... ... ... ... ... ... ... ... ... ... 153

The Hour of Delivery. By J. G. SWAYNE, M.D.... ... ... ... 174

Some Recent Developments of the Germ Theory, more particularly in Relation to the Treatment of Phthisis. By R. SHINGLETON SMITH, M.D., B.Sc., F.R.C.P. ... ... ... 225

CLINICAL RECORDS :—

A Case of Gouty Peripheral Neuritis. By F. W. JOLLYE ... ... 28

Notes from the Out-Patient Department. By W. J. PENNY ... 36

A Case of Suicidal Perforation of the Stomach by a Red-hot Iron. By LOCKHART STEPHENS ... ... ... ... ... ... 113

Notes of Out-Patient Cases. By J. MICHELL CLARKE, M.A., M.B. ... ... ... ... ...... ... ... ... ... ... ... ... 120

Page.

Notes on a Rare Case of Morbus Cæruleus. By P. Watson Williams, M.B. ... ... ... ... ... ... ... ... ... ... ... 183

Two Cases of Phthisis treated by means of Subcutaneous and Intra-Pulmonary Injections of Carbolate of Camphor. By R. Shingleton Smith, M.D., B.Sc. ... ... ... ... ... ... 185

Progressive Muscular Atrophy, with Recovery. By J. Michell Clarke, M.A., M.B. ... ... ... ... ... ... ... ... ... ... 195

Cases illustrating the Surgery of the Thyroid Body. By W. H. Harsant, F.R.C.S. ... ... ... ... ... ... ... ... ... ... 265

A Case of Raynaud's Disease. By Henry Waldo, M.D., M.R.C.P.... ... ... ... ... ... ... ... ... ... ... ... 272

Reviews of Books ... ... ... ... ... ... ... ... 51, 134, 198, 276

Notes on Preparations for the Sick ... ... ... 57, 140, 205, 291

Periscope ... ... ... ... ... ... ... ... ... ... ... 63, 145, 210, 293

Scraps ... ... ... ... ... ... ... ... ... ... ... ... 71, 151, 223, 299

FOUNDED 1831.

### H. W. CARTER & CO.'S
### HIGH CLASS MINERAL WATERS.

John Attfield, F.C.S., London; A. Hill, M.D., Birmingham; W.W. Stoddart, F.C.S., Bristol; F. F. Perkins, F.C.S., Exeter; Granville Sharpe, F.C.S., London; E. W. T. Jones, F.C.S., Wolverhampton; and other Experts, have testified to the purity of these celebrated Aerated Waters.

*The Original West of England Manufactory:*

OLD REFINERY, ST. PAUL'S, BRISTOL.

### Bristol Medico-Chirurgical Journal.

MARCH, 1888.

### CONTENTS.

*Cases for binding, price 7d. each, may be had of the Publisher,*
J. W. Arrowsmith, *Quay Street, Bristol.*

### *PUBLICATIONS RECEIVED.*

From F. Masias y Ca., Lima:
*Tratamiento de las Hemorragias por Retencion de la Placenta despues del Parto.* Por Anibal Fernandez Davila. 1887.

From Burdick & Taylor, Albany:
*Albany Medical Annals.* January, 1888.

From G. P. Engelhard & Co., Chicago:
*The Medical Standard.* December, 1887—February, 1888.

From John Carnrick, New York:
*The Dietetic Gazette.* January, 1888.

From P. Blakiston, Son & Co., Philadelphia:
*The Polyclinic.* December, 1887—February, 1888.

From Octave Doin, Paris:
*De l'Electricité comme Agent thérapeutique en Gynécologie.* Par le Docteur Paul F. Mundé. Traduit et annoté par le Dr. P. Ménière. 1888.
*Contribution à l'Etude de la Syphilis des Fosses Nasales.* Par le Dr. E. J. Moure. 1888.

From 23 Rue de la Monnaie, Paris:
*Le Naturaliste.* 15 Janvier, 1888.

From Baillière, Tindall, and Cox, London:
*Dental Caries and its Prevention.* By Henry Sewill. Second Edition. 1888.
*Manual for the Physiological Laboratory.* By Vincent Dormer Harris, M.D., and D'Arcy Power, M.B. Fourth Edition. 1888.
*Rabies and Hydrophobia.* By Surgeon-General C. A. Gordon, M.D., C.B. 1888.
*Laryngoscopy and Rhinoscopy.* By Prosser James, M.D. 1888.
*Aids to Obstetrics.* By Samuel Nall, M.B. Third Edition (n.d.)

From Cassell & Company, Limited, London:
*Diseases of the Breast.* By Thomas Bryant. 1887.

From J. & A. Churchill, London:
*The Treatment of Uterine Fibroids by Electrolysis.* By W. E. Steavenson, M.D. 1887.
*Biliousness.* By Adolphus E. Bridger, M.D. (Published by Henry Renshaw.)
*The Pathology of Intra-uterine Death.* By William O. Priestley, M.D. 1887.
*On Some Points in the Surgery of the Urinary Organs.* The Lettsomian Lectures. By Reginald Harrison. 1888.

From John Heywood, Manchester:
*Where Consumption is Bred in Manchester and Salford.* By Arthur Ransome, M.D. 1888.
*Infant Feeding and Infant Foods.* By J. McNaught, M.D. 1888.

From H. K. Lewis, London:
*Gout.* By Robson Roose, M.D. Fifth Edition. 1888.
*The Health of Children.* By Angel Money, M.D., B.S. 1888.

From E. & S. Livingstone, Edinburgh:
*Waters and Baths of Aix-les-Bains.* By Stanley M. Rendall, M.D. Second Edition. 1887.

From Longmans, Green, & Co., London:
*Lectures to Practitioners.* By W. T. Gairdner, M.D., and Joseph Coats, M.D. 1888.

From Young J. Pentland, Edinburgh:
*Student's Pocket Medical Lexicon.* By Elias Longley. 1888.
*Diseases of the Skin.* By W. Allan Jamieson, M.D. 1888.

### Exchange-List.

### AFRICA.

### CAPE COLONY.

*Weekly.*

*The South African Medical Journal.* Cathcart: W. Darley-Hartley.

### AMERICA.

### ARGENTINE REPUBLIC.

*Monthly.*

*Anales del Círculo Médico Argentino.* Buenos Ayres: M. Biedma.

### CANADA.

*Monthly.*

*Canada Medical and Surgical Journal.* Montreal: Gazette Printing Company.

### PERU.

*Monthly.*

*La Crónica Médica.* Lima: Carlos Prince.

### UNITED STATES.

*Weekly.*

*The Cincinnati Lancet-Clinic.* Cincinnati: 281 West 7th Street.

*The Journal of the American Medical Association.* Chicago: 65 Randolph Street.

*The Medical and Surgical Reporter.* Philadelphia: 115 South Seventh Street.

*The Medical Record.* New York: William Wood & Co.

*The New York Medical Journal.* New York: D. Appleton & Co.

*Fortnightly.*

*Philadelphia Medical Times.* Philadelphia: J. B. Lippincott & Co.

*The American Practitioner & News.* Louisville: John P. Morton and Company.

*Twice a month.*

*The Medical Age.* Detroit: George S. Davis.

*Monthly.*

*Index Medicus.* Detroit: George S. Davis.

*Journal of Cutaneous and Genito-Urinary Diseases.* New York: William Wood & Company.

*New Orleans Medical and Surgical Journal.* New Orleans: 33 Carondelet Street.

*Pacific Medical and Surgical Journal and Western Lancet.* San Francisco: Bonestell & Co.

*The American Journal of Ophthalmology.* St. Louis: J. H. Chambers & Co.

*Monthly.*

*The American Journal of the Medical Sciences.* Philadelphia: Lea Brothers & Co.

*The Medical Analectic.* New York: G. P. Putnam's Sons.

*The Obstetric Gazette.* Cincinnati: 281 West 7th Street.

*The Saint Louis Medical and Surgical Journal.* Saint Louis: 2622 Washington Avenue.

*The Therapeutic Gazette.* Detroit: George S. Davis.

*Yearly.*

*Annual of the Universal Medical Sciences.* Philadelphia: 1632 Chestnut Street.

*Transactions of the American Surgical Association.* Philadelphia: P. Blakiston, Son & Co.

*Occasionally.*

*Transactions of the New York Academy of Medicine.* New York: 12 West 31st Street.

### ASIA.

### INDIA.

*Monthly.*

*The Indian Medical Journal.* Calcutta: W. Newman & Co., Ld.

### JAPAN.

*Monthly.*

*The Sei-I-Kwai Medical Journal.* Tokio: Z. P. Maruya & Co.

### AUSTRALASIA.

### AUSTRALIA.

*Monthly.*

*The Australasian Medical Gazette.* Sydney: L. Bruck.

*The Australian Medical Journal.* Melbourne: Stillwell & Co.

### EUROPE.

### BELGIUM.

*Monthly.*

*Bulletin de l'Académie royale de médecine de Belgique.* Brussels: F. Hayez.

### BRITISH ISLES.

*Weekly.*

*British Medical Journal.* London: 429 Strand.

*The Hospital.* London: 38 Old Jewry.

*The Lancet.* London: 423 Strand.

*Monthly.*

*Annals of Surgery.* London: Baillière, Tindall & Cox.

*The Birmingham Medical Review.* Birmingham: Cornish Bros.

*The Journal of Laryngology and Rhinology.* London: R. Anderson & Co.

*The London Medical Recorder.* London: W. H. Allen & Co.

*The Medical Chronicle.* Manchester: John Heywood.

*The Practitioner.* London: Macmillan & Co.

*Edinburgh Medical Journal.* Edinburgh: Oliver and Boyd.

*The Glasgow Medical Journal.* Glasgow: Alex. Macdougall.

*The Dublin Journal of Medical Science.* Dublin: Fannin & Co.

*Quarterly.*

*The Asclepiad.* London: Longmans, Green, & Co.

*The British Gynæcological Journal.* London: Smith, Elder, & Co.

*Half-yearly.*

*The Liverpool Medico-Chirurgical Journal.* Liverpool: Medical Institution.

*The Retrospect of Medicine.* London: Simpkin, Marshall, & Co.

*Yearly.*

*The Medical Annual.* Bristol: John Wright & Co.

*The Year-Book of Treatment.* London: Cassell & Company, Limited.

*Year-Book of Scientific and Learned Societies.* London: C. Griffin & Co.

*Transactions of the Academy of Medicine in Ireland.* Dublin: Fannin and Company.

DENMARK.

*Weekly.*

*Hospitals-Tidende.* Copenhagen: Jacob Lund.

FRANCE.

*Three times a week.*

*Gazette des hôpitaux.* Paris: 4 Rue de l'Odéon.

*L'Union médicale.* Paris: 11 Rue de la Grange-Batelière.

*Weekly.*

*Bulletin de l'Académie de médecine.* Paris: G. Masson.

*Gazette médicale de Paris.* Paris: 8 Place de l'Odéon.

*Le Progrès médical.* Paris: 14 Rue des Carmes.

*Twice a month.*

*Gazette de Gynécologie.* Paris: O. Doin.

*Monthly.*

*Nouvelles Archives d'Obstétrique et de Gynécologie.* Paris: Félix Alcan.

*Revue mensuelle de Laryngologie, d'Otologie et de Rhinologie.* Paris: 8 Place de l'Odéon.

GERMANY.

*Twice a week.*

*Deutsche Medizinal-Zeitung.* Berlin: Eugen Grosser.

*Weekly.*

*Medicinische Bibliographie.* Leipzig: Breitkopf & Härtel.

HOLLAND.

*Weekly.*

*Nederlandsch Tijdschrift voor Geneeskunde.* Amsterdam: F. van Rossen.

ITALY.

*Daily.*

*La Riforma Medica.* Rome: Piazza S. Silvestro.

*Monthly.*

*Lo Sperimentale.* Florence: 35 Via degli Alfani.

NORWAY.

*Monthly.*

*Norsk Magazin for Lægevidenskaben.* Christiania: Th. Steens.

PORTUGAL.

*Weekly.*

*A Medicina Contemporanea.* Lisbon: José Antonio Rodrigues & Ca.

ROUMANIA.

*Monthly.*

*Spitalul.* Bucharest: Stefan Mihalescu.

RUSSIA.

*Weekly.*

*Vrach.* St. Petersburg: C. Ricker.

SPAIN.

*Weekly.*

*El Siglo Médico.* Madrid: Magdalena, 36, 2°

SWEDEN.

*Quarterly.*

*Nordiskt Medicinskt Arkiv.* Stockholm: P. A. Norstedt & Söner.

SWITZERLAND.

*Monthly.*

*Illustrirte Monatsschrift der ärztlichen Polytechnik und Centralblatt der orthopädischen Chirurgie.* Berne: Schmid, Francke & Co.

